# Impact of an excise tax on the consumption of sugar-sweetened beverages in young people living in poorer neighbourhoods of Catalonia, Spain: a difference in differences study

**DOI:** 10.1186/s12889-019-7908-5

**Published:** 2019-11-21

**Authors:** Miguel Ángel Royo-Bordonada, Carlos Fernández-Escobar, Lorena Simón, Belen Sanz-Barbero, Javier Padilla

**Affiliations:** 1 National School of Public Health, Institute of Health Carlos III, Sinesio Delgado, 8, 28029 Madrid, Spain; 2 National Center of Epidemiology, Institute of Health Carlos III, Madrid, Spain; 3 CIBER of Epidemiology and Public Health (CIBERESP), Madrid, Spain; 4 Primary Care Health Center Isabel II, Madrid, Parla Spain

**Keywords:** Taxes, Sugar-sweetened beverages, Spain, Catalonia

## Abstract

**Background:**

Sugar-sweetened beverage consumption is contributing to the obesity epidemic. On 28 March 2017, Catalonia enacted a law levying an excise tax on sugar-sweetened beverages for public health reasons. The purpose of this study is to assess the impact of the tax on the consumption of sugar-sweetened beverages in Catalonia (Spain).

**Methods:**

Before-and-after study to assess changes in the prevalence of consumption of sugar-sweetened beverages among 1929 persons aged 12 to 40 years residing in low-income neighbourhoods of Barcelona (intervention) and Madrid (control). Beverage consumption frequency was ascertained via a validated questionnaire administered during the month prior to the tax’s introduction (May 2017) and again at 1 year after it had come into force. The effect of the tax was obtained using Poisson regression models with robust variance weighted using propensity scores.

**Results:**

While the prevalence of regular consumers of taxed beverages fell by 39% in Barcelona as compared to Madrid, the prevalence of consumers of untaxed beverages remained stable. The main reason cited by more than two-thirds of those surveyed for reducing their consumption of sugar-sweetened beverages was the increase in price, followed by a heightened awareness of their health effects.

**Conclusions:**

The introduction of the Catalonian excise tax on sugar-sweetened beverages was followed by a reduction in the prevalence of regular consumers of taxed beverages.

## Background

The prevalence of obesity among the Spanish population has risen progressively in recent decades [1], reaching figures of over 20% in adults [2] and around 10% in children [3]. High body mass index is the leading cause of disease burden in Spain, being responsible for more than 10% of disability-adjusted life years, essentially due to its association with cardiovascular diseases, several types of cancer, and metabolic and endocrine diseases [4]. In Spain, prevalence of obesity displays an inverse socio-economic gradient, both at a macro level, with per capita income in the Autonomous Regions, and at a micro level, with families’ educational level and socio-economic status [2, 5].

Globally-speaking, consumption of sugar-sweetened beverages (SSBs) ranks high among the many causes of obesity [6], and this is equally true of the Spanish population, where an increment in soft drinks consumption of 100 ml was associated with a 0.21 kg/m^2^ increase in BMI [7]. As SSBs contain energy in liquid form which generates low satiety levels and a compensatory response inadequate to counteract excess calories, they may alter the balance between energy intake and expenditure [8]. Furthermore, intake of SSBs triggers high blood sugar peaks, which favour insulin resistance and diabetes [9], and is associated with the development of hypertension and hyperlipidaemia [10]. Consumption of one SSB per day is estimated to increase cardiovascular risk, both fatal and non-fatal, by 20% [11].

According to the 2014 European Health Survey, 35.9% of the Spanish population aged 15 years and over were regular consumers of soft drinks [12]. It has been estimated that 0.6% of all deaths in Spain are attributable to such consumption, with a total of 30 annual deaths per million adults [13]. Consumption is higher among adolescents, with a mean intake of soft drinks, fruit juices and drinks of over 450 mL/day [14], which accounts for more than 6% of total calorie intake [15]. As with obesity, here in Spain SSB consumption displays an inverse gradient with socio-economic level. According to data drawn from the 2012 National Health Survey, the daily percentage of SSB consumers in the lower socio-economic stratum of the Spanish population is two to three times higher than that in the high-income bracket, among adults and children alike [3, 16].

At the World Health Organisation (WHO) European Ministerial Conference on Nutrition and Noncommunicable Diseases held in Vienna in 2013, the Ministers of Health of the European Region Member States undertook to lend impetus to the application of economic tools to promote healthy dietary habits [17]. The European Food and Nutrition Action Plan advocates that consideration be given to food-supply-chain incentives in the form of subsidies and taxation [18], and the WHO Committee Report on eliminating childhood obesity recommends levying a tax on SSBs [19]. In the wake of the initiative launched by the city of Berkeley (California) and countries such as Finland, France, UK, South Africa and Mexico, other cities across the USA and countries around the world started introducing excise taxes on SSBs in 2017 and 2018 [20]. Following this trend [21], on 28 March 2017 Catalonia enacted a law levying an excise tax on SSBs for public health reasons [22]. While fruit drinks, sports drinks, tea and coffee, energy and vegetable drinks, sugar-sweetened milk drinks, shakes, soft drinks, and flavoured water are all subject to this tax, natural fruit juices, fermented milk drinks and drinking yoghurts are exempt. The tax in Catalonia is unique as it was designed through legislation to be fully passed through to prices and includes two ‘tiers’. Although a similar UK soft drinks tiered levy was introduced in april 2018, it was designed to be passed through to manufacturers [23]. The law renders the tax payable by the consumer at a rate of 8 centimes per litre for beverages with a sugar content of 5 to 8 g per 100 mL and 12 centimes per litre for beverages with higher sugar content, which is expected to raise the price between 10 and 20% on average, depending on sugar content and container size, rates similar to those applied in other countries with similar statutory measures [24].

Evidence of the effectiveness of excise taxes on SSBs is growing fast, with observed reductions on frequency of consumption in Philadelphia [25] and low-income areas of Berkley [26], and decreased sales in Mexico [27], Barbados [28] and Chile [29]. A recent systematic review of real-word studies concluded that SSB taxes are effective in reducing SSB purchases an dietary intake, suggesting a greater effet for volumetric taxes with sugar thresholds [30]. Besides price mechanisms via which a tax influences consumption, a singalling effect has been described in adults who were aware of the SSB tax [31]. This study evaluated the impact of the tax on the consumption of SSBs in Catalonia, using two samples of Barcelona townspeople (pre- and post-taxation) and, by way of a control group, two comparable samples of Madrid townspeople of similar characteristics, providing the first evidence on consumption for a tiered SSB tax designed to be fully passed through to prices. Moreover, we examined subjects’ degree of knowledge of the tax, self-perceived changes in consumption patterns, and the reasons cited by them to account for these changes in the Barcelona post-tax sample.

## Methods

### Design

We conducted a before-and-after, quasi-experimental study, with a control group for comparison purposes, using repeated application of a non-alcoholic beverage consumption survey to assess the effect of the tax on SSBs in Catalonia. The pre-taxation survey was conducted in April 2017, the month before the tax came into force (1 May 2017), and the post-taxation survey was conducted 1 year later, to avoid confounding due to seasonal variations.

### Study subjects and selection of the sample

The study covered young persons (age range 12–40 years) of both sexes residing in districts with the lower index of disposable family income in Barcelona (Nou Barris and Sant Andreu) [32] and Madrid (Usera and Puente de Vallecas) [33], a population group with a higher SSB consumption on which the tax’s foreseeable impact would be greater [34]. A requirement for inviting adolescents aged 12 to 15 years to participate in the study was that they be accompanied by an adult relative.

In each city, a number of sampling points were selected in busy areas of the study districts, such as markets, bus, rail and underground stations, and the environs of shopping malls or football grounds. Interviewers positioned at the sampling points invited all passers-by to take part in a non-alcoholic beverage consumption survey, asking for their oral consent (and, in the case of adolescents aged 12 to 15 years, the consent of an accompanying family member). The following were excluded from study: any person outside the age range or not residing in the study district; and any person who presented with a disease or intellectual-type disability that prevented him/her from properly supplying the information required. The main goal of the study was only revealed to the participants once they had answered the questionnaire, in order to avoid any risk of bias potentially associated with knowledge of said goal when reporting their consumption patterns.

**Fig. 1:**
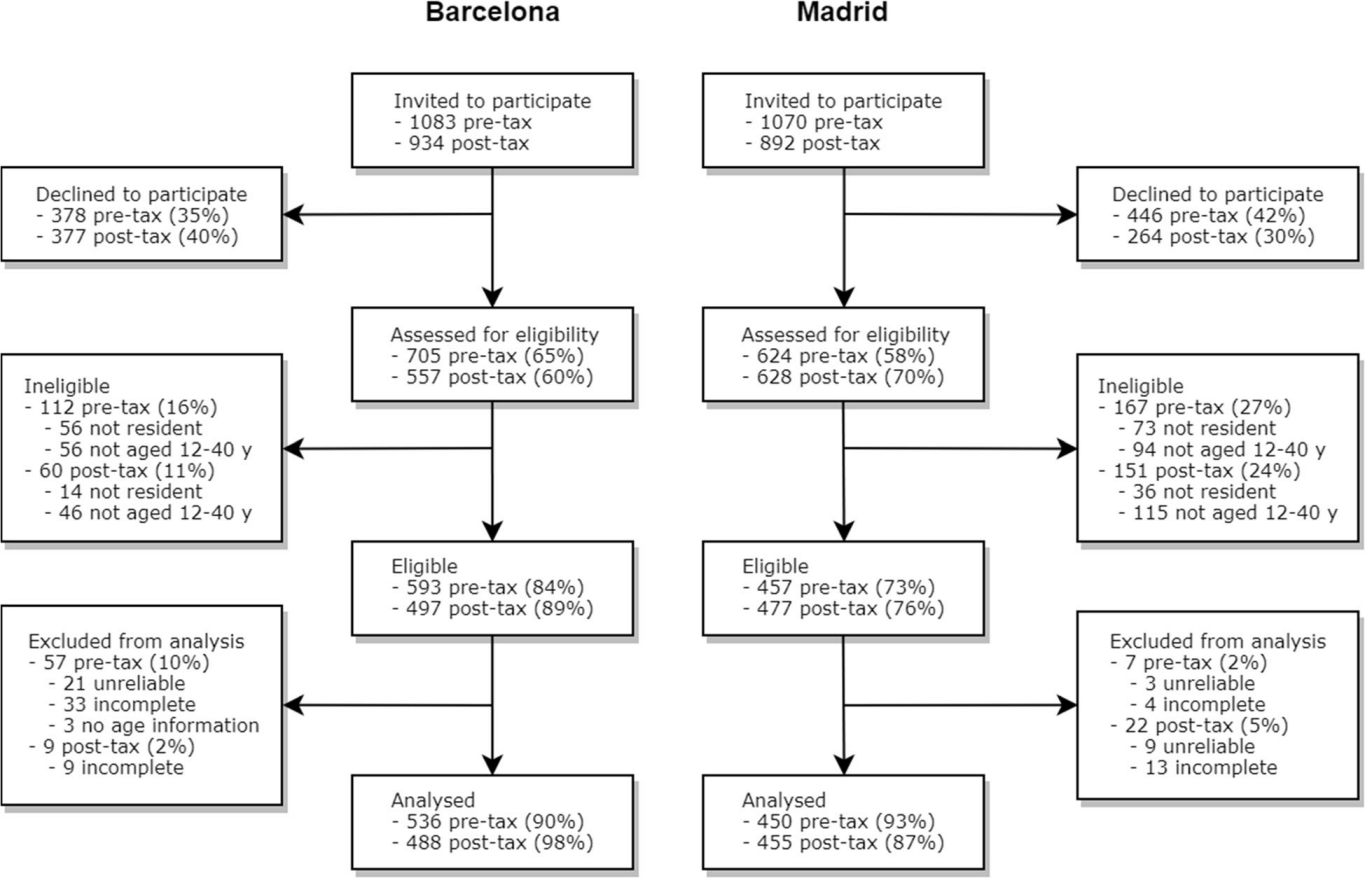
Flow chart showing participants in the pre-tax (2017) and post-tax (2018) surveys in low-income neighbourhoods of Barcelona (intervention) and Madrid (control)

A total of 3979 persons were invited to participate; of these 1465 (36.8%) rejected the invitation (Fig. 1). The participation rate was somewhat lower in the pre-taxation survey (61.7%) than in the post-taxation survey (64.9%), with a variable distribution by city. Of the 2514 persons who agreed to participate, 459 (18.3%) were excluded for not residing in the study districts or falling outside the age range, with this percentage being higher in Madrid than in Barcelona both pre- and post-tax. Of the 2055 persons eligible, 95 (4.6%) were excluded for failing to give reliable answers, due to inadequate comprehension of the questions or to giving incomplete responses, with missing values in questions pertaining to age, consumption of beverages, or most of the covariates. The highest percentage of exclusions was observed in the Barcelona pre-taxation survey, with a figure of 10%. The final study sample totalled 1929 persons, 986 in the pre-taxation and 943 in the post-taxation surveys.

### Data-collection and study variables

SSB consumption data were gathered by purpose-trained interviewers who administered a survey, which included a section with socio-demographic variables (age, sex, nationality, educational level and occupational status), and a non-alcoholic beverage consumption questionnaire, previously validated and adapted for the Spanish population [35]. The structure of the questionnaire was adapted to ask subjects about their regular consumption (at least once per week) of each type of beverage from the statutory categories, namely, taxed beverages (soft drinks, fruit drinks and energy drinks) and untaxed beverages (sugar-free soft drinks, fruit juices and drinking yoghurts). Flavoured water and vegetable drinks were also included in the latter category because the added sugar content of the products available on the market in no case exceeded 5 g/100 mL, the taxation threshold. Sports drinks and sugar-sweetened milk drinks are shown in a separate category, due to the fact that they include some varieties not subject to the tax by reason of their lower sugar content. For each type of beverage included in the questionnaire, subjects were asked whether it was consumed daily or weekly and, if so, whether or not the regularly consumed beverage was an own-brand product.

In the Barcelona post-taxation survey, an additional section was included at the end of the questionnaire, in which subjects were asked if they knew of the tax’s existence and if they had changed their SSB consumption patterns after the tax’s entry into force, whether by reducing the amount consumed or totally or partially replacing it with an untaxed or own-brand beverage. Lastly, those who reported that they had changed their consumption habits were asked whether the reasons for the change were connected with the increase in the price of SSBs, enhanced awareness of their health effects, or some other reasons.

In addition, we collected information on the price of nine of the most popular beverages in different formats (large and small; with and without sugar; soft, fruit, and energy drinks; registered and own-brand beverages), sold in the seven main supermarkets present both in Madrid and in Barcelona, covering more than 75% of the beverages market share [36]. As beverage prices in supermarkets are the same on-site than online, but do vary depending on your place of residence, data prices were collected online by including the postal code (Madrid vs. Barcelona) in the supermarket web pages, in the months of March 2017 and March 2018.

### Statistical analysis

We conducted a descriptive study of the socio-demographic data collected in each of the surveys (pre- and post-taxation) for each of the cities (Barcelona and Madrid) and calculated the corresponding prevalences of regular consumption of each type of beverage. Similarly, we calculated the frequency of attitudes to and opinions expressed about the tax in the survey. Comparison of socio-demographic variables was performed using Pearson’s Chi^2^ test for qualitative variables and Student's t test for quantitative variables.

The measure of association used to assess the effect of the tax was the ratio of post- to pre-tax prevalences of regular consumption with its 95% confidence interval, obtained using weigthed Poisson regression with robust variance. To obtain the weights, we first defined the variable Group with four categories, resulting from the combination of city and period (1: Barcelona pre-tax, 2: Barcelona post-tax, 3: Madrid pre-tax, and 4: Madrid post-tax). Then, we fitted a multinomial logistic regression to predict Group as a function of the socio-demographic variables included in the survey (age, sex, nationality, educational level and occupational status), resulting in 4 propensity scores (the probability of being in each of the 4 groups) for each individual. The weigths were then created in such a way that each of the groups was weigthed to be similar to the intervention city in the pre period (Barcelona pre-tax). To do that, for each individual, we divided the probability to being in group 1 by the probability to being in the group he or she actually was [37]. In such way, individuals in other groups received a weigth proportional to their probability of being in group 1 relative to the probability of their being in the group they were actually in. Then, we fitted the weigthed Poisson regression models with the interaction term between period (pre-tax: 2017/post-tax: 2018) and city (Barcelona/Madrid), thereby obtaining the adjusted post- versus pre-tax ratio of prevalences of regular consumption in Barcelona, with the change in the prevalence of consumption in Madrid across the same period taken as reference. As 260 subjects (13.5%) had missing values in educational level, a new category of this variable was created so as to be able to include them in the multivariate regression analyses. Identical analyses were performed to assess the possible effect of the tax on the percentage of own-brand consumers among regular drinkers of taxed beverages.

The tax’s impact on the product’s end cost to the consumer was obtained by comparing the differences in prices between Madrid and Barcelona main supermarkets before and after the tax’s implementation, by container size. All analyses were performed using the STATA statistical software programme [38].

## Results

Table 1 shows the characteristics of the sample. The mean age of participants was 28.7 years, with this being higher in the post-taxation samples. Almost half of the Madrid post-taxation participants were of foreign nationality, double the figure of the remaining subsamples. The Barcelona post-taxation sample had 22.2% of participants with no formal or primary education only, versus figures of over 40% for the rest of subsamples. The percentage of students was higher among the pre-taxation participants in both cities, while the highest and lowest percentages of unemployed were seen among the post-taxation participants of Madrid and Barcelona respectively.

**Table 1:** Socio-demographic characteristics of the pre-tax (2017) and post-tax samples (2018) in low-income neighbourhoods of Barcelona (intervention) and Madrid (control)

N	Pre-tax		*p*-value ^a^	Post-tax		*p*-value ^a^
	Barcelona	Madrid		Barcelona	Madrid	
	536	450		488	455	
Age, years						
Mean (SD)	27.9 (7.8)	26.7 (7.9)	0.015	29.8 (7.2)	30.5 (7.2)	0.109
Women, %	52.2	47.2	0.121	41.0	58.5	< 0.001
Nationality, %			0.535			< 0.001
Spanish	77.2	78.8		81.6	52.1	
Other	22.8	21.2		18.4	47.9	
Educational level, %	(*n* = 278)	(*n* = 449)	0.086	(*n* = 488)	(*n* = 454)	< 0.001
Less than primary	5.4	10.9		3.1	6.6	
Primary	38.5	37.2		19.1	45.8	
Secondary	38.9	36.1		45.3	37.4	
University	17.3	15.8		32.6	10.1	
Occupational status, %	(*n* = 531)	(n = 449)	0.009	(*n* = 486)		< 0.001
Gainfully employed	39.9	50.6		68.1	59.3	
Unemployed	16.4	14.7		10.3	20.2	
Housework	5.8	4.9		3.1	5.9	
Student	37.8	29.8		18.5	14.5	

^a^ Student’s t test for age and Pearson’s Chi^2^ test for the remaining variables

**Fig. 2:**
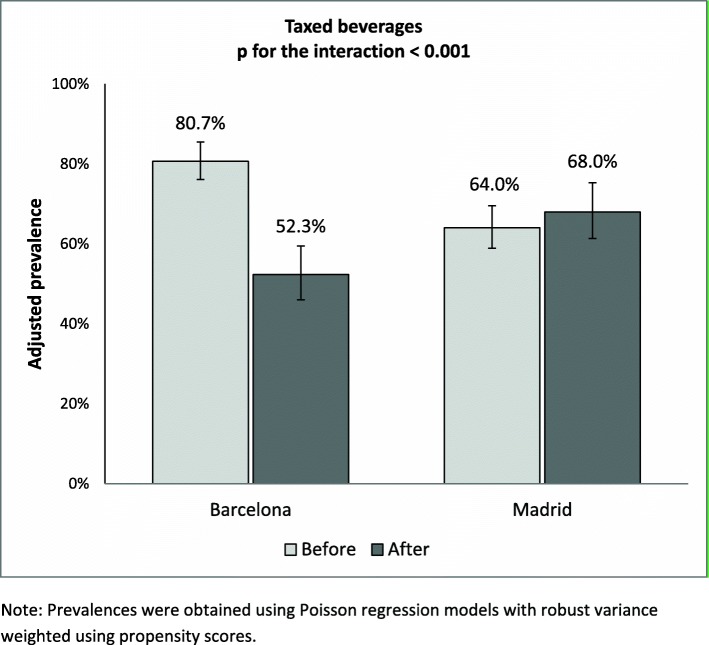
Adjusted prevalences of consumption of taxed beverages before (2017) and after (2018) taxation in Barcelona (intervention) and Madrid (control)

Following the tax’s introduction, the adjusted prevalence of regular consumers of taxed beverages decreased by 35.2% in Barcelona and increased by 6.2% in Madrid (Fig. 2). Table 2 shows the pre- and post-tax prevalence of regular consumers in both cities and the adjusted effect of the tax in Barcelona for each type of beverage, taking the change observed in Madrid over the same period as reference. Pre-tax prevalence was higher in Barcelona than Madrid both for taxed and untaxed beverages (76.9% vs. 63.8 and 80.8% and 68.2%, respectively), whit the highest prevalences being for soft drinks (50% vs. 47.1%). Whereas the post-tax prevalence of consumers of all three types of taxed beverages decreased in Barcelona, it increased in Madrid, with the single exception of energy drinks which showed no variation. Taking Madrid as reference, the prevalence of regular consumers of taxed beverages in Barcelona fell by 39% (*p* < 0.01), with a 29% fall in the prevalence of consumers of soft drinks, an 70% fall in that of fruit drinks and 77% fall in that of energy drinks (*p* < 0.01). The percentage of consumers of sports drinks and sugar-sweetened milk drinks decreased in both cities post-tax, though the fall was sharper in Barcelona, resulting in a reduction in the prevalence of such consumers with respect to Madrid of 58 and 66% respectively (*p* < 0.01).

**Table 2:** Prevalence of regular consumption of taxed and untaxed beverages in low-income neighbourhoods of Barcelona and Madrid before (2017) and after (2018) taxation, and ratio of post- to pre-tax prevalence in Barcelona with respect to Madrid

	Barcelona (*n* = 1024)			Madrid (*n* = 905)			Ratio of post- to pre-tax prevalence in Barcelona relative to Madrid (95% CI)^a^
	Pre-tax % (95% CI)	Post-tax % (95% CI)	Post- to pre-tax difference (%)	Pre-tax % (95% CI)	Post-tax % (95% CI)	Post- to pre-tax difference (%)	
Taxed beverages							
Soft drinks	50.0 (45.8–54.2)	46.3 (41.9–50.8)	−3.7 (−7.4)	47.1 (42.5–51.7)	54.3 (49.7–58.9)	7.2 (15.2)	0.71 (0.54–0.93)
Fruit drinks	42.9 (38.7–47.1)	11.3 (8.5–14.1)	−31.6 (−73.7)	20.2 (16.5–23.9)	27.0 (22.9–31.1)	6.8 (33.7)	0.30 (0.19–0.49)
Energy drinks	36.8 (32.7–40.8)	14.3 (11.2–17.5)	−22.4 (−61.0)	22.2 (18.4–26.1)	22.6 (18.8–26.5)	0.4 (1.9)	0.23 (0.14–0.38)
Total	76.9 (73.3–80.4)	52.0 (47.6–56.5)	−24.8 (−32.3)	63.8 (59.3–68.2)	69.0 (64.7–73.3)	5.2 (8.2)	0.61 (0.50–0.74)
Other taxed beverages^b^							
Sports drinks	26.9 (23.1–30.6)	9.2 (6.6–11.8)	− 17.6 (− 65.7)	15.8 (12.4–19.2)	11.9 (8.9–14.9)	−3.9 (−24.8)	0.42 (0.22–0.81)
Sugar-sweetened milk drinks	30.0 (26.1–33.9)	5.9 (3.8–8.0)	- 24.1 (−80.2)	28.9 (24.7–33.1)	20.2 (16.5–23.9)	− 8.7 (− 30)	0.34 (0.19–0.64)
Untaxed beverages^c^	80.8 (77.4–84.1)	83.6 (80.3–86.9)	2.8 (3.5)	68.2 (63.9–72.5)	76.5 (72.6–80.4)	8.3 (12.1)	1.03 (0.90–1.20)
Taxed own-brand beverages ^d^	43.4 (38.6–48.3)	35 (29.1–40.9)	−8.4 (−19.4)	37.3 (31.7–42.9)	50.0 (44.4–55.6)	12.7 (34.1)	0.89 (0.62–1.26)

^a^ The ratio of prevalences was obtained from the interaction between year (pre-tax: 2017/post-tax: 2018) and city (Barcelona/Madrid) in Poisson regression models with robust variance adjusted for age, sex, educational level, nationality and occupational status

^b^ Energy drinks and sugar-sweetened milk drinks are shown in a separate category, due to the inclusion of certain varieties not subject to the tax by reason of their lower sugar content

^c^ Includes: fruit drinks, soft drinks, flavoured water, vegetable drinks and juices, and drinking yoghurts

^d^ Prevalence of use of own-brand products among consumers of taxed beverages

**Fig. 3:**
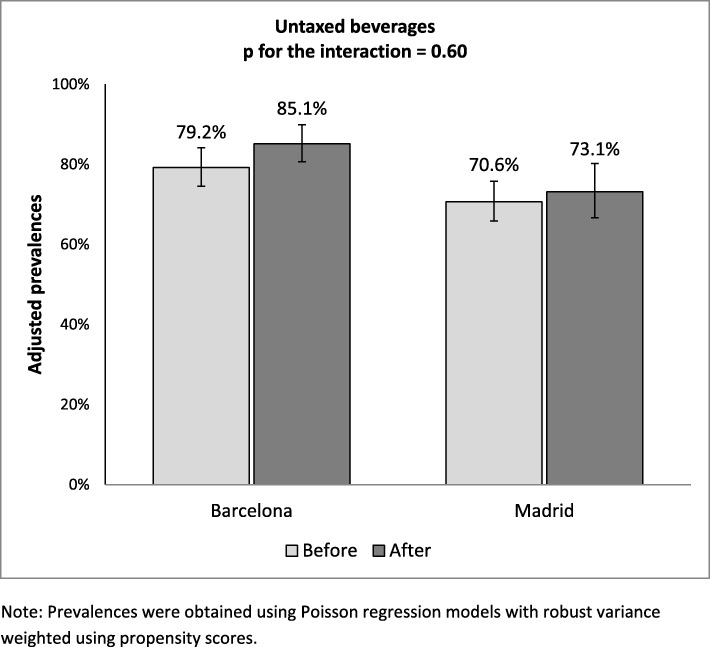
Adjusted prevalences of consumption of untaxed beverages before (2017) and after (2018) taxation in Barcelona (intervention) and Madrid (control)

After the tax, the prevalence of consumers of untaxed beverages increased by 7.4% in Madrid and 4% in Barcelona (Fig. 3), though neither of these differences proved statistically significant, which translated as a lack of effect when it came to comparing the change in prevalence between the two cities (Table 2). Similarly, among regular drinkers of taxed beverages, the percentage of own-brand consumers remained stable in Barcelona as compared to Madrid.

A total of 83.4% of the post-taxation participants in Barcelona knew of the existence of the tax on SSBs, and 37.4% of these reported having changed their consumption habits of non-alcoholic beverages as a consequence (Table 3): 77% of those who changed their habits reported having reduced their consumption, 13.8% reported having partly or totally replaced the taxed beverages with other untaxed or own-brand drinks, and the remainder reported having combined reduction in consumption with some degree of substitution. The main reason given for changing consumption patterns was the increase in price in 75% of the sample, with this being the exclusive reason in 67.1%; similarly, while 30.3% reported that the change was due to enhanced awareness of the health effects of SSBs, this was cited as the exclusive reason by 22.4%.

**Table 3:** Knowledge of the tax on sugar-sweetened beverages, changes in consumption, and reasons for the change reported by the 455 participants in the post-tax sample (2018) in low-income neighbourhoods of Barcelona

	N (%)
Knows of the existence of the tax	407 (83.4%)
Reports change in consumption^a^	152 (37.3%)
Direction of change in consumption ^b^	
Reduction in consumption	117 (77.0%)
Replacement by untaxed beverages	9 (5.9%)
Replacement by own-brand beverages	12 (7.9%)
A combination of the above	14 (9.2%)
Reason for change in consumption ^b^	
Increase in price	102 (67.1%)
Health awareness	34 (22.4%)
Increase in price and health awareness	12 (7.9%)
Others	4 (2.6%)

^a^ Percentage calculated with respect to the 407 participants who knew of the existence of the tax

^b^ Percentage of the 152 participants who reported having changed their consumption of beverages as a result of the tax’s introduction

As compared to Madrid, mean prices rose by 8.3% for drinks in small-sized containers (less than 1 l) and 17.5% for the rest. The greatest price rises were observed for own-brand soft drinks in large-sized containers, with an increase of 26.3%.

## Discussion

This is the first study to assess the impact of the Catalonian excise tax on consumption of SSBs by young residents of low-income neighbourhoods in Spain. While the prevalence of regular consumers of taxed beverages fell by 39% in Barcelona as compared to Madrid, no change was observed in the prevalence of consumers of untaxed beverages. Among regular drinkers of taxed beverages, the percentage of own-brand consumers remained unchanged in Barcelona as against Madrid. After the tax’s introduction, the main reason cited by just over two-thirds of those surveyed in the Barcelona sample for reducing their consumption of SSBs was the rise in price, followed by a heightened awareness of their health effects.

This paper provides the first evidence of the effect on SSB consumption for a tiered SSB tax design designed through legislation to be fully passed through to consumers. In our study, more than two thirds of those who reported a reduction in SSB consumption stated the increase in price as a reason for change, like in Hungary [39]. Hence, being affordability a major driver of SSB purchasing behaviours [40], while targeting manufacturers may increase public support for the tax, like in the UK [23], its potential effect on SSB consumption can be comprised if the tax is not fully passed to consumers. Our data on SSB prices show that the tax was passed through to consumers, with 8.3% for small-sized containers and 17.5% for large-sized containers, using the change in prices in Madrid over the same period as reference for comparison purposes. The greatest price rise, 26.3%, was for taxed own-brand beverages in large-sized containers, as expected being the tax charged at a fixed amount per volume of liquid. Health awareness was the second reason for change, by almost one third of those who reported a reduction in SSB consumption, corroborating a signalling effect suggested in previous studies [31, 39]. The fact that the tax was justified by health reasons and implemented after a long public debate in the mass media [41], has probably contributed to the high levels of public awareness of both the tax and the health risks of SSBs.

In accordance with the results of previous reviews [30, 42], price elasticity for SSBs is around − 1.00, though this magnitude is higher among the young, socio-economically underprivileged population, such as that of our study. Analysed by type of beverage, elasticity is greater for fruit and sports drinks. In a New Zealand study, elasticity for the two lower quintiles of socio-economic level was − 2 to − 3 for soft drinks and − 3 to − 5 for energy drinks [43]. Consistent with that, the effect magnitude in our study was higher for fruit drinks, energy drinks and sports drinks, an outcome that was likewise observed in Berkeley [26], using the same methodology, and in Mexico [44], using an SSB-sales time series. The results of the assessment in Mexico show that the magnitude of the effect is negligible in the first months post-taxation and that it then becomes steadily more intense until reaching a peak at 12 months, coinciding with the period during which we carried out our assessment. In contrast, consumption of untaxed beverages increased in Mexico during the first months and then fell to pre-tax levels at 1 year of the tax’s introduction, a result consistent with the lack of effect observed by us, at 1 year post-tax, for untaxed beverages.

According to the Spanish Health Survey, the prevalence of soft drink regular consumers in 2016 and 2017 was higher in the population of all ages of Catalonia than in that of Madrid (43% vs. 35,9%) [45], like in our study (50% vs. 47,1%). As expected, the figures were higher in our study, with a sample of young people from deprived areas. In Hungary, the only European country to have conducted a formal assessment of the tax on SSBs, using a population sample over the age of 18 years, the prevalence of regular consumers of soft drinks at 1 year of the tax’s introduction fell by 20%, a figure lower than the 29% observed in our study, with younger people living in poorer neighbourhoods, though this rose to 25% in the group with the lowest educational level [39]. This is consistent with larger reductions in taxed bevegares observed in Mexico among high purchasers [46]. Again, the effect in Hungary was of a greater magnitude for energy drinks, but not as much as in Barcelona. This difference may be due in part, to the fact that the impact is greater in the younger population [24], such as that of our study. However, it could also be due to the fact that the percentage of pre-tax consumers was higher in Catalonia (36.8%) than in Hungary (22%), which suggests a possible floor effect, something that should be analysed in future studies, since the prevalence of regular consumers at 1 year of the tax’s introduction was very similar (14.3 and 16% respectively).

Two main arguments are levelled against the tax on SSBs [47]. The first is that it is a regressive, discriminatory measure: nonetheless, the higher prevalence of consumption in low-income Spanish families [3] and the intensity of response observed in this study, which targeted neighbourhoods with the lowest income per capita in Madrid and Barcelona, may well translate as future health benefits for the more underprivileged population [48] if, as observed in low-income neighborhoods of Berkley after 3 years of the SSBs tax, the reduction in frequency of consumption persist over time [49]. The fall in frequency of regular consumers of SSBs in the Spanish child and adolescent population during the economic crisis was 2 to 3 times sharper in the low-income stratum, among which the percentage of daily consumers fell by 50%, an impact similar to that detected in our study [3]. The second argument against the tax is that any effect may be offset by increased consumption of similar products, such as own-brand beverages or less healthy products. In Denmark, the tax on fats caused customers of the more expensive supermarkets to shift to discount stores [24]. In our study, although 7.9% of those who changed their consumption habits in Barcelona reported that they had partially or totally replaced the taxed beverages with own-brand beverages, the percentage of consumers of taxed beverages who opt for own-brand varieties remained unchanged.

### Limitations

Owing to the quasi-experimental design, causal relationships cannot be established with certainty, due to the possibility of unmeasured or residual confounding. The method of recruitment and the wheather varying conditions may explain the fairly significant socio-demographic differences between groups. To control for these confounding factors, we used a control group and adjusted the regression models for socio-demographic variables using propensity scores. Our model also relies on the common trends assumption [50]. Using monthly data from the Spanish Food Purchases Panel [36], similar trends were observed and no violation of the common trends assumption was found in soft drink sales for both cola drink taxed (*p* = 0.97) and untaxed (*p* = 0,87) bevegares (Additional files 1 and 2). Prices data should also be taken with caution, as they are not representative of local or regional supermarkets, food and grocery stores, bars and restaurants. Another limitation, which hinders comparisons with other studies, is the fact that we only measured the frequency of consumption and not the amount consumed. Even so, our results are internally consistent and in line with those of studies on price elasticity and the tax’s differential impact for each type of beverage in other countries where similar measures have been applied. Although the choice of a sample of young adults from low-income neighbourhoods limits our capacity to extrapolate the results to the general population, in studies using broader-based samples the tax has been observed to affect all socio-economic strata, though the magnitude of the effect is less in the highers strata [48]. The presence of 13.5% of participants with missing values in the educational level variable had no influence on the results, since the category created to include such subjects in the regression models did not prove predictive of SSB consumption, and the effect estimators were very similar when these subjects were included. Lastly, sports drinks and sugar-sweetened milk drinks include some product with sugar levels below the tax threshold, and these categories were thus analysed separately, though the results show a tax effect in the same direction as that of the remaining taxed beverages.

## Conclusions

At 1 year of its introduction, the Catalonian excise tax on SSBs has brought about an important fall in the prevalence of regular consumers of taxed beverages. Future studies will have to assess whether this change is maintained over time or whether it becomes more marked as has been observed in Mexico, and measure, not only the frequency of consumers, but also the amounts consumed, in order to have a more accurate estimator of the tax’s impact. In the interim, our results, along with the remaining scientific evidence on the subject, would justify the extension of the measure to the rest of Spain for public health reasons.

## Supplementary information

**Additional file 1. **Monthly cola drink purchases per capita in Madrid (A) and Barcelona (B) before the tax from January 2013 to Abril 2017. The figures show the evolution in Spanish Food Purchases Panel sample (*N* = 12,000) of sugary cola drink (taxed beverage), from January 2013 to Abril 2017 (before the tax), in Barcelona (A) and Madrid (B). We can not reject the common trend null hypothesis, as the t-test for regression time coefficients difference with homogenous variances gives a *p* value of 0.97.

**Additional file 2. **Monthly cola light drink purchases per capita in Madrid (A) and Barcelona (B) before the tax from January 2013 to Abril 2017. The figures show the evolution in Spanish Food Purchases Panel sample (*N* = 12,000) of sugary cola light drink (untaxed beverage), from January 2013 to Abril 2017 (before the tax), in Barcelona (A) and Madrid (B). We can not reject the common trend null hypothesis, as the t-test for regression time coefficients difference with homogenous variances gives a *p* value of 0.87.

## Data Availability

The datasets used and analysed during the current study are available from the corresponding author on reasonable request.

## Abbreviations

SSBs: Sugar-sweetened beverages
WHO: World Health Organisation
