# Supplementary material for: Impact of an excise tax on the consumption of sugar-sweetened beverages in young people living in poorer neighbourhoods of Catalonia, Spain: a difference in differences study
Source: BMC Public Health. 2019 Nov 21;19:1553. doi: 10.1186/s12889-019-7908-5 (PMC6873539; doi:10.1186/s12889-019-7908-5)
Supplement: Supplementary file 1 — Additional file 1. Monthly cola drink purchases per capita in Madrid (A) and Barcelona (B) before the tax from January 2013 to Abril 2017. The figures show the evolution in Spanish Food Purchases Panel sample (N = 12,000) of sugary cola drink (taxed beverage), from January 2013 to Abril 2017 (before the tax), in Barcelona (A) and Madrid (B). We can not reject the common trend null hypothesis, as the t-test for regression time coefficients difference with homogenous variances gives a p value of 0.97. [file 12889_2019_7908_MOESM1_ESM.docx]

**A**

**B**

**Additional file 1.** Monthly cola drink purchases per capita in Madrid (A) and Barcelona (B) before the tax from January 2013 to Abril 2017.
